# Bridging the gap: Can single session interventions help enhance mental health treatment delivery for young people in Australia?

**DOI:** 10.1177/00048674241271964

**Published:** 2024-08-16

**Authors:** Matthew Thompson, Marcela Radunz, Tracey D Wade, Ryan P Balzan

**Affiliations:** 1 Flinders University Institute for Mental Health and Wellbeing, Flinders University, Bedford Park, SA, Australia; 2 Blackbird Initiative, Flinders University, Adelaide, SA, Australia

## The problem

There is currently a major discrepancy between need and access to mental health services in Australia and across the world. Self-reports indicate that less than half of young people in Australia who need treatment receive appropriate care for mental health issues (Brennan et al., 2021). Practical barriers to help-seeking exist, including cost, accessibility, and availability of services. Psychological treatments are often expensive, require multiple sessions with a registered mental health professional, based in urban centres and limited in availability, with waitlists for less-expensive services such as headspace or university clinics exceeding multiple months. These barriers remain despite substantial investment in the mental health services by the Australian government. As such, treatment options are needed that are both cost-effective and readily available to consumers at the time they need it.

## Single-Session Interventions

Single-session interventions (SSIs) are structured interventions that are deliberately delivered in a way that assumes a single encounter with a clinic, provider or programme (Schleider et al., 2020). These services may be therapist-led or self-guided and can be utilised as stand-alone treatments or as augmentation to more traditional therapeutic approaches. Although SSIs may be completed on multiple occasions or as adjuncts to intentionally longer-term care, they are designed such that any individual session holds potential to yield some degree of positive, meaningful change at a time when people are considering change (Schleider et al., 2020). While engaging in multiple SSIs or multiple sessions of online interventions would likely lead to more desirable therapeutic outcomes, the median retention rate of therapeutic apps after 15 days is just 4% (Baumel et al., 2019). This indicates that, despite the potential benefit of multi-session, low-resource interventions, the average user is not willing to dedicate sufficient time to complete these as intended. As such, the ability to produce change in a single interaction with a therapeutic intervention is a significant advantage. While this may not fully alleviate symptoms, there is growing evidence for various SSIs in reducing symptoms of a multitude of disorders in both clinical and subclinical populations, especially in youth. Specifically, a meta-analysis by Schleider and Weisz (2017) showed SSIs significantly reduced symptomatology in young people with psychiatric problems with group effect sizes (ES) averaging 0.41 (compared to no treatment) and 0.14 (compared to active controls). These benefits have been found from a range of SSIs including those that are online and self-guided, suggesting the potential for SSIs that require minimal resources and are highly scalable (Schleider et al., 2020). SSIs can also be used flexibly, targeted to specific needs, such as (1) early intervention (for populations at risk but who do not have a mental health disorder), (2) providing a ‘taster plate’ for people considering seeking treatment, (3) keeping people engaged in change while they are waiting for services, (4) helping people engage in early treatment change, a critical factor for good outcome in mental health treatment (Chang et al., 2021), and (5) augmenting treatment.

## SSIs and treatment outcomes

SSIs are not a silver bullet that will replace the need for longer-term, psychological support. However, by utilising SSIs as a tool to help improve treatment delivery, treatment outcomes and availability may be improved. As longer waitlists are known to reduce the likelihood of an individual following through with treatment, if readily available SSIs are provided to those on waitlists, they may help people stay engaged and improve their likelihood of accessing ongoing treatment.

Further to increasing the likelihood of accessing other therapies, SSIs may also be able to improve treatment outcomes. Preliminary evidence from an ongoing clinical trial run by the Flinders University Service for Eating Disorders (FUSED) shows that over 50% of those offered either a self-guided growth mind-set or behavioural activation SSI while on a waitlist for treatment achieved more than a 30% reduction in dietary restraint over a 2-week period, prior to commencing therapy. This early decrease in key symptoms is encouraging given that early change in eating disorder symptomatology is the most robust predictor of good outcome in treatment (Chang et al., 2021). As such, the use of SSIs may increase the likelihood of a good treatment outcome and, given the lower symptom severity upon commencing therapy, may allow individuals to achieve results in fewer sessions. Ultimately, this could reduce the amount of contact clients need with the mental health system, reducing the strain on the system and allowing for a greater availability of resources.

## Limitations and future directions

SSIs could improve engagement with psychological care. However, a few key areas of research must be addressed before they become more widely used.

1. What is the best utility of SSIs? At present, SSIs have most commonly been tested as stand-alone interventions. While they have regularly shown utility in producing small to medium improvements in some symptomology, they are unlikely to be able to produce such substantial changes that an individual no longer needs therapy. As such, SSIs may be more useful for those on the waitlist for ongoing therapy or as an adjunct to those already receiving therapy to more quickly improve symptoms and reduce their time spent in therapy. As such, more research looking at acceptability and the short- and long-term efficacy of SSIs as part of larger therapeutic programmes would be beneficial.
2. Does acceptability of SSIs in research trials translate to uptake in the real world? Few research studies have been conducted outside the controlled conditions of studies and without incentivisation. Additional research in services such as FUSED could be mutually beneficially to services and researchers alike, as services have the potential to see more clients and reduce their waitlists, while researchers can gain data on SSIs in the real world, from a readily available pool of participants.
3. What are the longer-term outcomes of SSIs? There is little evidence on whether potential improvements in outcomes due to stand-alone SSIs or a combination of traditional therapies and SSIs are maintained in the long-term (e.g. follow-up up to 1 year after treatment). The benefit of SSIs easing the current burden on the mental health system is diminished if individuals experience symptom recurrence and need to re-engage with the mental health system. Without solid evidence for this key piece of information, the real-world benefits of SSIs for service availability remains unknown.

## Conclusion

SSIs present a promising avenue for enhancing treatment delivery and increasing the availability of psychological help, particularly for youths. SSIs require few resources and are highly scalable with the potential to be widely distributed and available with little to no wait times. SSIs may also help reduce the waitlists for other, traditional psychological therapies by reducing the number of sessions required with a psychologist and increasing the availability of resources in the mental health system. The preliminary efficacy of SSIs in improving symptoms and risk factors associated with multiple disorders is promising. However, before we can readily deploy these interventions as a part of our primary response to mental health, we must ensure that we understand how SSIs function in the real-world settings and how they can be best utilised to augment existing mental health treatment approaches.
